# The growth modulating effects of tether tension on vertebral growth are biphasic: a study of posterior vertebral body tethering (pVBT) in a novel kyphotic porcine model

**DOI:** 10.1007/s43390-025-01168-y

**Published:** 2025-08-20

**Authors:** Matthew A. Halanski, Brittney Kokinos, Ellen Leiferman, Minhao Zhou, Yousuf Abubakr, Max Twedt, Cameron Jeffers, David Bennett, Susan Hamman, Jennifer Frank, Melanie E. Boeyer, Grace D. O’Connell, Thomas Crenshaw

**Affiliations:** 1 Division of Orthopedics and Sports Medicine, Phoenix Children’s Hospital, Phoenix, AZ USA; 2 University of Arizona College of Medicine-Phoenix, Phoenix, AZ USA; 3 Departments of Orthopedics and Rehabilitation and Animals Sciences, University of Wisconsin-Madison, Madison, WI USA; 4 Mechanical Engineering, University of California-Berkeley, Berkeley, CA USA; 5 Orthopedics and Rehabilitation, University of Nebraska Medical Center, Omaha, NE USA; 6 Missouri Orthopaedic Institute, University of Missouri School of Medicine, Columbia, MO USA

**Keywords:** Posterior vertebral body tethering (VBT), Growth modulation, Physis, Animal model

## Abstract

**Purpose:**

To measure the effects of posterior vertebral tethering (pVBT) on disc pressure and the effect of tether tension on growth modulation in the hyperkyphotic swine model, and to use computational modeling to predict growth modulation in scenarios unable to be tested in the animal model.

**Methods:**

Swine were divided into non-operative control, single-level apical pVBT, or multi-level posterior pVBT groups. Pulsed fluorochrome labeling was used to measure regional vertebral growth rates, digital radiographs to assess changes in vertebral alignment, and pressure transducers to measure intervertebral disc pressures. Finite element analysis (FEA) was performed to simulate tether-mediated disc space correction.

**Results:**

Kyphotic swine had significantly greater angular kyphosis than control swine at 11- and 13-weeks, and deformities increased from 2 to 5 months of age. At 2-weeks post-operative, high-tension single level tethering resulted in significantly greater growth modulation than low-tension (53 ± 43% vs − 1 ± 15%, *p* = 0.03) or non-operative controls (*p* = 0.01), however, at 2–4 weeks, growth modulation was normalized between the low and high tensioned cohorts (14 ± 11% vs 10 ± 10%, *p* = 0.6). The FEA predicted that growth plate stress distributions *worsen* as the average disc height post-realignment is decreased.

**Conclusion:**

Increased tether tension results in more effective early growth modulation in the young flexible spine without increasing disc pressure, however, these tension-related benefits are transitory as growth modulation becomes load-independent with time. Computational modeling predicted that in the less flexible spine, vertebral growth may be arrested rather than modulated.

**Supplementary Information:**

The online version contains supplementary material available at 10.1007/s43390-025-01168-y.

## Introduction

Vertebral body tethering (VBT) is currently used as an alternative to spinal fusion for the treatment of scoliosis in skeletally immature children and adolescents. The early widespread enthusiasm for the technique has waned as short-term success has been reported in only 59–74% of cases [1, 2] due, in part, to limited postoperative growth modulation [3–6]. While popularized as an alternative surgical treatment for scoliosis, vertebral growth modulation has been used to treat sagittal plane deformities. Lowe et al. demonstrated the feasibility of the technique to decrease kyphosis and vertebral wedging in the ovine model [7]. Clinically, the technique was found to be successful in modulating vertebral growth in a young patient with kyphoscoliosis [8]. Recently, an adaptation of the technique has been described to correct Scheuermann’s kyphosis [9]. Unlike the previous studies [7, 8], the authors report improved sagittal alignment without premature fusion**.**

Historically, nearly all preclinical development of VBT and other vertebral growth modulation strategies have relied on use of the inverse approach [2, 10–19] which inferred the effectiveness of a growth modulation technique by its ability to create a deformity, or a two-stepped approach [20–26], in which a deformity was surgically created and then surgically corrected. These previous studies failed to use a natively deformed spine to study growth modulation techniques [27]. We have previously described a hyper-kyphotic porcine model, produced through maternal and neonatal vitamin D restriction in diets with surplus calcium and phosphorus [28], that exhibits a progressive kyphotic deformity during growth [28], resembling Scheuermann’s disease in humans [29–32]. These hyper-kyphotic swine can be predictably produced as offspring of conventional, crossbred dams fed hypovitaminosis D diets during gestation and lactation with additional modifications of diets fed to the offspring for a 3- to 4-week post-weaning phase [33]. The conventional crossbred genetic lines have well-characterized nutritional requirements with defined growth and reproductive developmental traits. We have employed the use of this non-surgically induced spinal deformity model to better understand deformity progression and growth modulation in the natively deformed spine. This novel, large-animal model facilitates the investigation of surgical variables not easily or ethically testable in children. The purpose of the current study was to assess the feasibility of posterior VBT (pVBT) to modulate vertebral growth in this non-surgically created large animal model to better understand the effects of tether tension on growth modulation.

## Methods

### Experimental overview

This study was reviewed and approved by the University of Wisconsin Institutional Animal Care and Use Committee. Hyper-kyphotic and control swine were bred and produced as previously described [28]. Thirteen kyphotic and 10 control animals served as non-operative controls to assess baseline deformity (lateral Cobb angle) and regional growth rates at approximately 11- and 13-weeks of age. These animals also served as untreated controls to our operative cohorts. Three of the non-operative kyphotic pigs were followed out past 5 months of age to document curve progression over time. To measure apical disc pressure changes in response to increasing posterior tether load, 7 swine (*N* = 4 control and *N* = 3 kyphotic) were utilized in non-recovery experiments. A separate cohort of kyphotic animals were used for recovery surgeries, with 8 undergoing single level (2 vertebrae) posterior tethering to study the early effects of High (*N* = 4) and Low (*N* = 4) tension and 8 undergoing multi-level (6 vertebrae) High (*N* = 4) or Low (*N* = 4) tension posterior tethering to assess the later effects of tether tension.

### Surgical procedures

#### Non-recovery and fresh cadaveric posterior tethering and disc pressure testing

In the prone position, a posterior midline incision and sub-periosteal dissection were performed over the apical 4-levels of kyphotic spines and 5.5 mm cannulated screws (Orthopediatrics, Warsaw IN) were placed bilaterally in the vertebral pedicles. A lateral approach was then made just ventral to the paraspinal muscles and transverse processes. Blunt dissection was used to come down on the lateral surface of the vertebral body. Fibers of the psoas were swept away, and the apical disc was identified via lateral radiography. A Gaeltech pressure transducer was advanced into the nucleus pulposus (NP) and entry voltage recorded, and pressures calculated. Stainless-steel cables (Atlas Cables, Medtronic, Minneapolis, MN) were placed posteriorly around each pair of screws and a hand-held load cell was attached to the cables as they were progressively tensioned with the pressure transducer left in the disc space. Lateral radiographs were taken to document the change in vertebral alignment and disc pressure voltage was recorded.

#### Recovery single-level posterior tethering

The apex of the deformity was confirmed on radiographs and the Wiltse style approach was used to place 5.5 × 28–32 mm cannulated screws (OrthoPediatrics, Warsaw, IN) into the pedicles and advanced to leave room for the tether cable to pass between the screw head and lamina. A pair of Medtronic Atlas tether cables were placed around the screw of the cranial vertebra and caudal vertebra on each side of the spine. The cables were tensioned using the handheld load cell to place Low (5N) or High (25.6N) tension on the vertebrae. Cables were crimped and the incisions were sutured, and the final lateral and dorsal–ventral radiographs were taken. The animals were given their first bone label and recovered. Two weeks post-operative, animals were given a second bone label and harvested.

#### Recovery multi-level posterior tethering

A similar surgical approach and procedure were performed in the multi-level cohort; however, pairs of screws were placed at six contiguous vertebral levels with pairs of cables spanning over five intervertebral discs in each animal**.** Tensioning of the cables was performed starting at the apex and proceeding outward. Animals were recovered and weekly lateral radiographs performed. An initial cohort of two animals (*N* = 1 low and *N* = 1 high tension) were followed for 6 weeks to determine the estimated time of correction/overcorrection of the deformity. The remaining 6 animals (*N* = 3 low and *N* = 3 high tension) received fluorochrome labels at 2- and 4 weeks post-operatively to assess how tether tension affected apical growth modulation at these later time points.

### Outcome measures

#### Cobb angle measurement

Standard portable digital radiography was used (XJet, C.F.D. Devices, Scanzorosciate, Italy). Screening Cobb angle measurements were made using the XJet proprietary PACS software. Quantitative measurements were made using either Sante dicom viewer (Santesoft, Nicosia, Cypress) or Microdicom (Sofia, Bulgaria). As the instrumentation in the multi-level tether cohort did not encompass the entire kyphotic deformity, junctional kyphosis developed adjacent to the instrumented levels. To determine the effect of the tether tension on growth modulation and minimize the confounding effects of the adjacent junctional kyphosis, Cobb measurements in this cohort were measured within the instrumented levels only.

#### Pulsed fluorochrome labeling, regional growth rate measurements, and growth modulation

Pulsed fluorochrome labeling was performed by administering Alizarin Red 12–14 days prior to harvest and Oxytetracycline on the day of harvest. Vertebrae were coronally sectioned into ~ 3 mm thick slabs utilizing an Isomet Precision saw (Buehler Isomet 2000; Lake Bluff, IL), and the entire spinal segment was visualized using a Nikon NiE upright microscope (Nikon Instruments; Melville, NY) set-up for epifluorescence at 4X magnification. Regional growth rate (µm/day) was determined by measuring the distance between fluorochrome labels using a custom validated image analysis program [34] and dividing this distance by the time between fluorochrome label administration. Growth rates were reported for the entire physis, anterior 1/4, middle 1/2, and posterior 1/4 of the vertebrae, as well as the rate of growth under the unossified anterior epiphysis. This regional division was based on the anatomic structure of the disc/epiphyseal structure and the different biomechanical properties annulus and NP [35–41]. As the annulus of the disc attaches to the anterior and posterior ~ 1/4 of the vertebra and the NP occupies the central ~ 1/2 of the disc space, the authors felt that the different mechanical properties of the annulus and NP would transmit tether forces differently to each region of the adjacent growth plates.

For comparisons between non-kyphotic and kyphotic non-operative controls, growth rates of the apical vertebra (the sum of proximal and distal physis of the same vertebra) are presented. When analyzing the effects of tether tension, the growth rates on either side of the tethered disc space are presented (the sum of the distal physeal growth of the proximal vertebra and the proximal physeal growth rate of the distal vertebra).

In this manuscript, we have defined growth modulation as % *Growth Modulation*:

$$\frac{\mathrm{Anterior} \frac{1}{4} \text{growth rate} - \mathrm{Posterior} \frac{1}{4} \text{growth rate}}{\text{Total mean vertebral growth rate}}\times 100$$

This equation evaluates the differences in growth rates (anterior versus posterior) and normalizes the differences by the overall vertebral physeal growth rates to account for different rates of growth in individual animals. Thus, in our kyphotic model, a positive % Growth Modulation is indicative of (therapeutic) growth modulation (anterior > posterior growth) whereas negative % growth modulation is indicative of worsening kyphosis.

#### Finite element modeling to predict growth modulation

Our animal testing was limited by the flexibility of the young spines. To assess the effects of tethering a stiffer spine, a previously validated bone-disc-bone finite element model (FEM) of a bovine bone-disc-bone motion segment was used to assess the effects of tether-mediated changes in disc height during correction (FEBio.org) [39, 40, 42, 43]. Disc geometry was modified to represent porcine anatomy (height: 7 mm, radius: 15 mm). The FEM included separate components for the NP, inner and outer annulus fibrosus, cartilage endplates, growth plates, and vertebral bodies. Triphasic mixture theory was employed to account for the tissue deformation associated with proteoglycan-driven tissue swelling and fluid flow.

Boundary and loading conditions were defined such that a motion segment with a 10° disc deformity was corrected with tethering by simulating the bending and translation motion needed to re-align an individual disc level. Bending and translation loading conditions were applied through the intervertebral disc. Model-predicted intradiscal pressure and tether load were compared to our experimental kyphotic porcine data and with values reported in the literature [42, 44, 45].

**Fig. 1:**
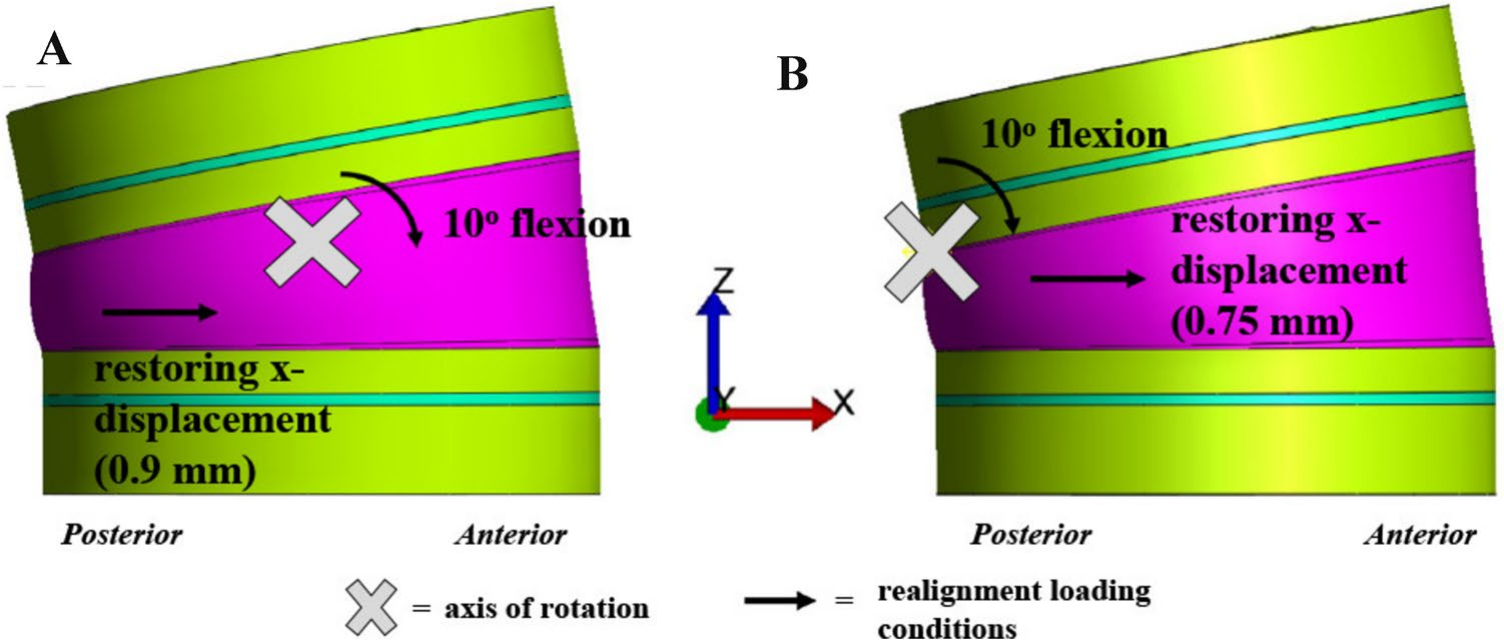
**a** Representative realignment loading configurations and axes of rotation for flexible and **b** stiff disc space tethering finite element models

To simulate behavior observed with younger flexible spines, the axis of rotation was defined to be located at the center of the disc (Fig. 1A—“X”). In this case, tethering would result in an increase anterior (concave) disc height, and decrease the posterior (convex) disc height, while the mid-disc height remained constant. A second simulation was conducted to simulate tethering correction in a stiffer spine by shifting the location of the axis of rotation to the anterior (concave) edge of the disc (Fig. 1B—“X”). In this simulation, the anterior (concave) disc height remained constant, while the disc height in the middle and posterior (convex) portion of the disc decreased with tethering.

Stresses in the growth plate were evaluated and compared between the two simulations. Increased tensile stresses on the growth plate have been shown to increase longitudinal growth, whereas 0.5 MPa, or greater of compressive stress leads to growth arrest [44, 46]. Therefore, 0.5 MPa of compression stress in the cartilage endplate was used as the threshold for predicting vertebral growth arrest. The growth plate stress distribution was compared to growth plate growth modulation data measured in bovine caudal vertebrae [46], and predictions of vertebral growth modulation made from these stress distributions based on changes in mid-discal height.

### Statistical analysis

Mean values and standard deviations are presented for measured variables. Paired Student t-tests were employed to compare measurements with continuous variables within the same animal, whereas unpaired *t*-tests were used to compare measurements between subjects. *p*-value ≤ 0.05 was denoted as statistically significant.

## Results

### Non-operative controls

Kyphotic swine had greater angular kyphosis than control swine at 11- and 13-weeks (47.8° ± 2° vs 38.3° ± 4°, *p* = 0.001 and 60.6° ± 9° vs 40° ± 3°, *p* = 0.001). Mean apical disc pressures lying prone on the exam table were greater in the non-kyphotic swine than kyphotic swine at 11- and 13-weeks, as was overall weight (Table 1). Mean vertebral growth rates were similar between kyphotic and non-kyphotic swine, and both demonstrated increased posterior to anterior growth (Table 1). Mean deformities increased in kyphotic animals, from 44.2° ± 2.7° at 2 months to 76.4° ± 7.6° at 5 months of age (*p* = 0.008) with a mean vertebral growth rate of 65 ± 25 μm/day.

**Table 1:** Comparison of kyphotic and normal non-operative controls

	~ 11-week-old			~ 13-week-old		
	Kyphotic (*N* = 5)	Control (*N* = 5)		Kyphotic (*N* = 5)	Control (*N* = 5)	
	Mean ± STD	Mean ± STD	*p*-value	Mean ± STD	Mean ± STD	*p*-value
Age	10.7 ± 0.3	11.4 ± 0.3	0.005	12.5 ± 0.3	13.3 ± 0.3	0.002
Sagittal Cobb°	47.74 ± 2.9	38.8 ± 3.8	0.0016	60.1 ± 8.8	40 ± 3.4	0.0014
Apical Cobb°	8.06 ± 0.5	6.3 ± 1.1	0.0054	15.2 ± 6.1	4.5 ± 1.1	0.0051
Apical discs (× 2) pressure combined (kPa)	77.0 ± 15.9	122.4 ± 37.5*	0.0018	59.5 ± 14.2	115.5 ± 24.5	0.0002
Weight gain final 2 weeks (kg)	8.86 ± 0.6	11.8 ± 0.8	0.0001	9.9 ± 1.3	12.2 ± 0.7	0.0042
Final weight (kg)	28.26 ± 2.2	41 ± 6.0	0.0011	41.3 ± 6.7	55.2 ± 3.5	0.0017
Mean apical vertebral growth rate (μm/day)	178 ± 21	188 ± 11	0.16	173 ± 28	172 ± 29	0.49
Mean anterior (1/4) vertebral growth rate	174 ± 16	172 ± − 6	0.43	163 ± 21	145 ± 24	0.12
Mean posterior (1/4) vertebral growth rate	191 ± 21	202 ± 12	0.16	195 ± 34	20 ± 35	0.35

Radiographs for Cobb measurements were made in a standard prone position on a table with hind limbs brought forward. Mean disc pressures were taken from the two most apical disc in each cohort

^*^Measurements are from 7/10 discs due to technical issues

### Tether tension, lateral Cobb angle, and disc pressure

**Fig. 2:**
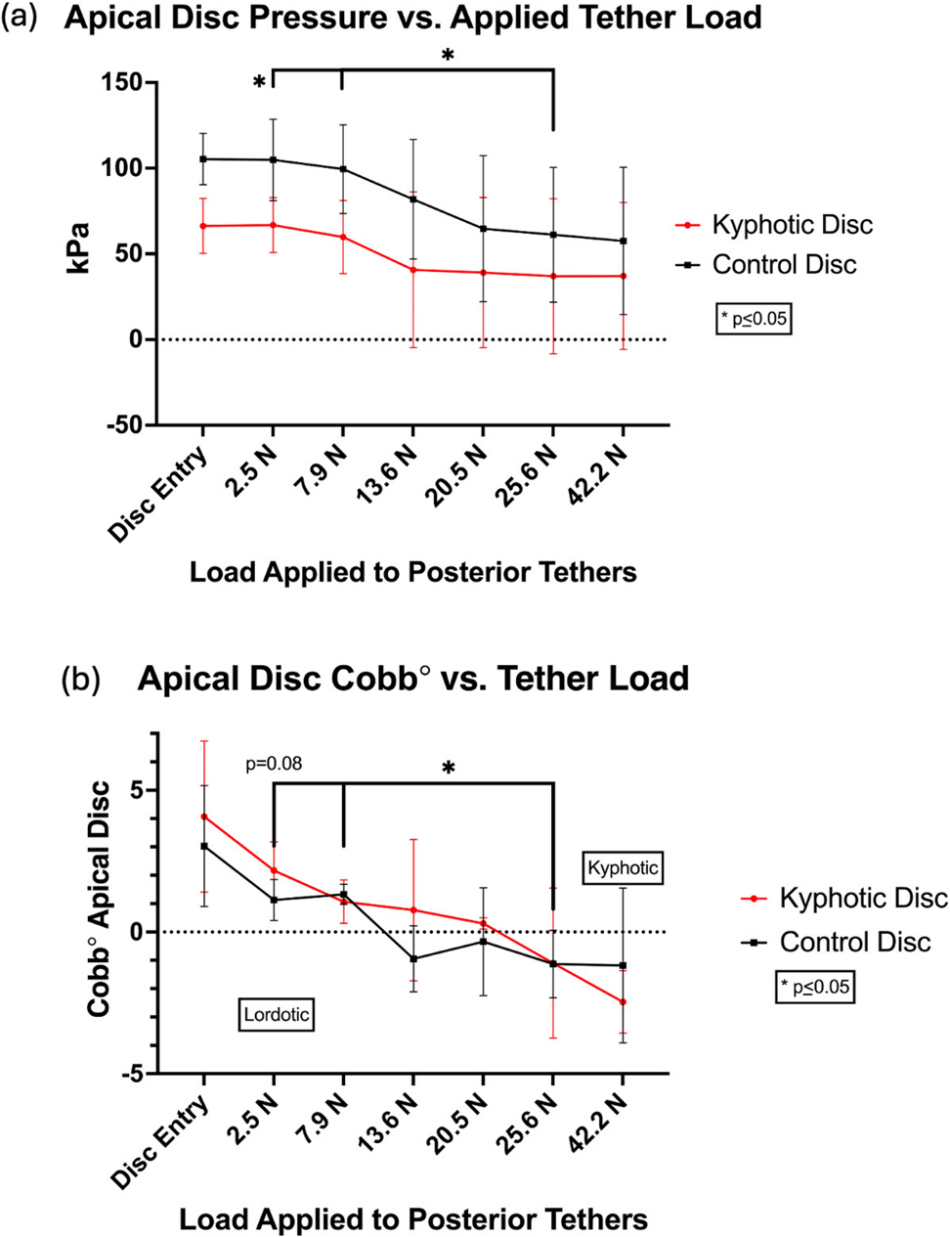
**a** Increasing tether load resulted in a reversal of the apical cobb angle from kyphotic to lordotic. **b** Disc pressure was found to decrease as this change in vertebral angulation occurred

Increasing posterior tether load in 10–11-week-old non-recovery swine resulted in reversal of apical disc deformity from kyphotic to lordotic in both control and kyphotic animals (Fig. 2a). The change in disc angle was accompanied by decreased apical disc pressures, however, this reduction only reached significance in the control spines (99 ± 25 vs 61 ± 39 kPa, *p* = 0.02) (Fig. 2b).

### Single-level tethering

**Fig. 3:**
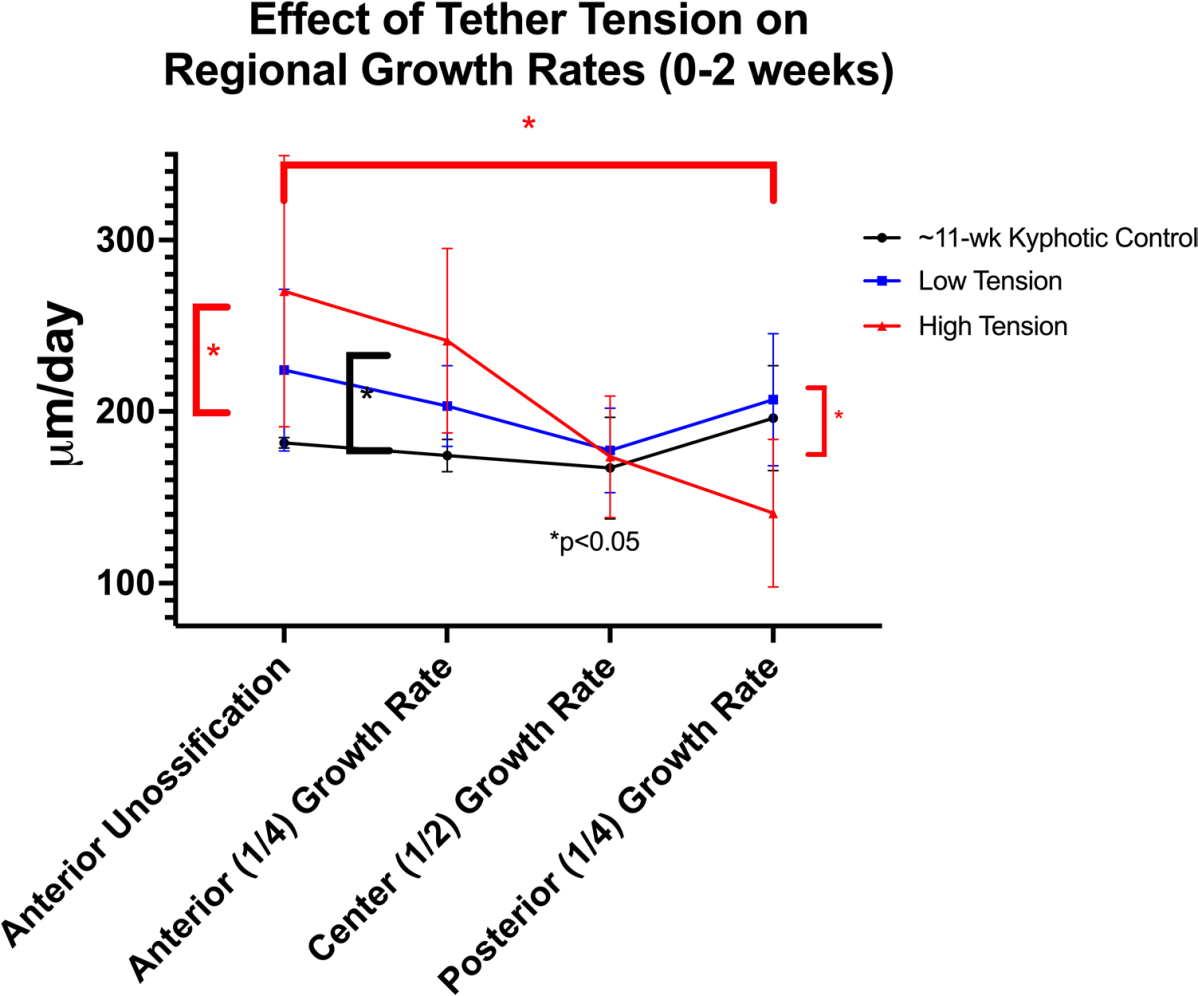
Comparison of the tether vertebral space regional growth rates of the single-tethered kyphotic swine with the non-operative kyphotic swine during initial 2 weeks following surgery

Two weeks post-operatively, the operative cohorts were slightly older than their non-operative controls (11.2 ± 0.1 vs 10.7 ± 0.3 weeks, *p* < 0.001). Higher tension resulted in *increased* anterior (241 ± 54 vs 157 ± 12 μm/day, *p* = 0.04) and *decreased* posterior (141 ± 43 vs 198 ± 38 μm/day, *p* = 0.001) growth rates compared to adjacent untethered physes (internal controls). High-tension, single-level tethering resulted in significantly greater growth modulation (53 ± 43%) than lower tether tension (− 1 ± 15% *p* = 0.03) or non-operative controls (− 11 ± 17% *p* = 0.01) (Fig. 3).

### Multi-level tethering

**Fig. 4:**
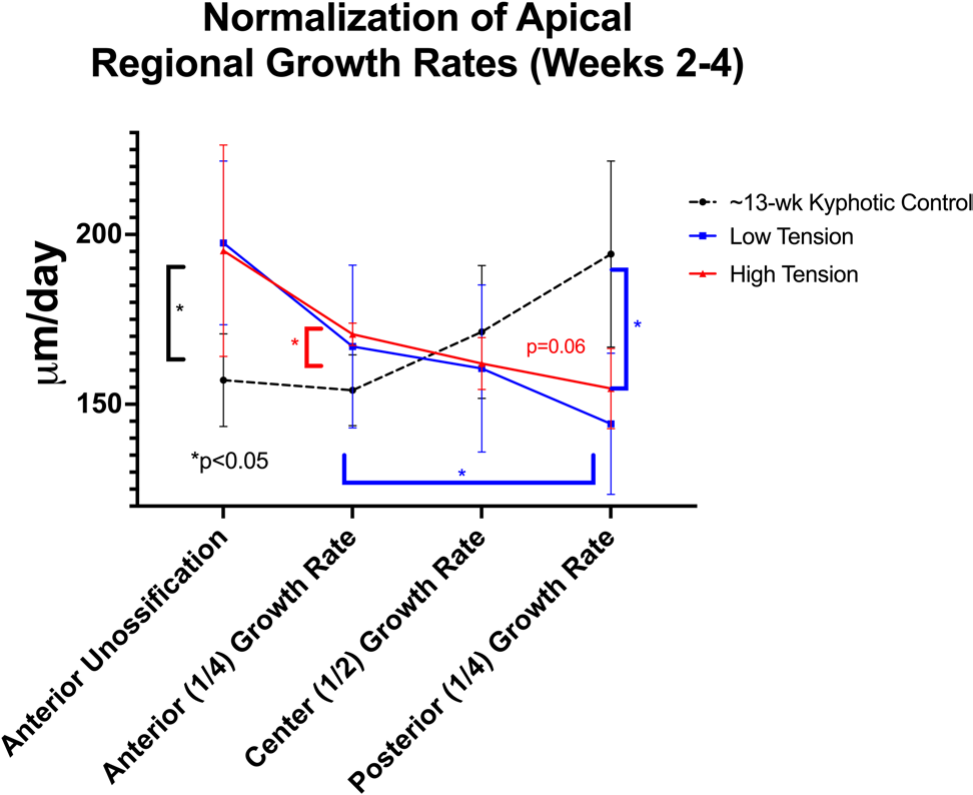
The central tethered vertebral space regional growth rates in the high and low tensioned cohorts during weeks 2–4 post op. Notice the normalization of the growth modulation and blunted effect of the tethering when compared during the initial post-op period (Fig. 3)

**Fig. 5:**
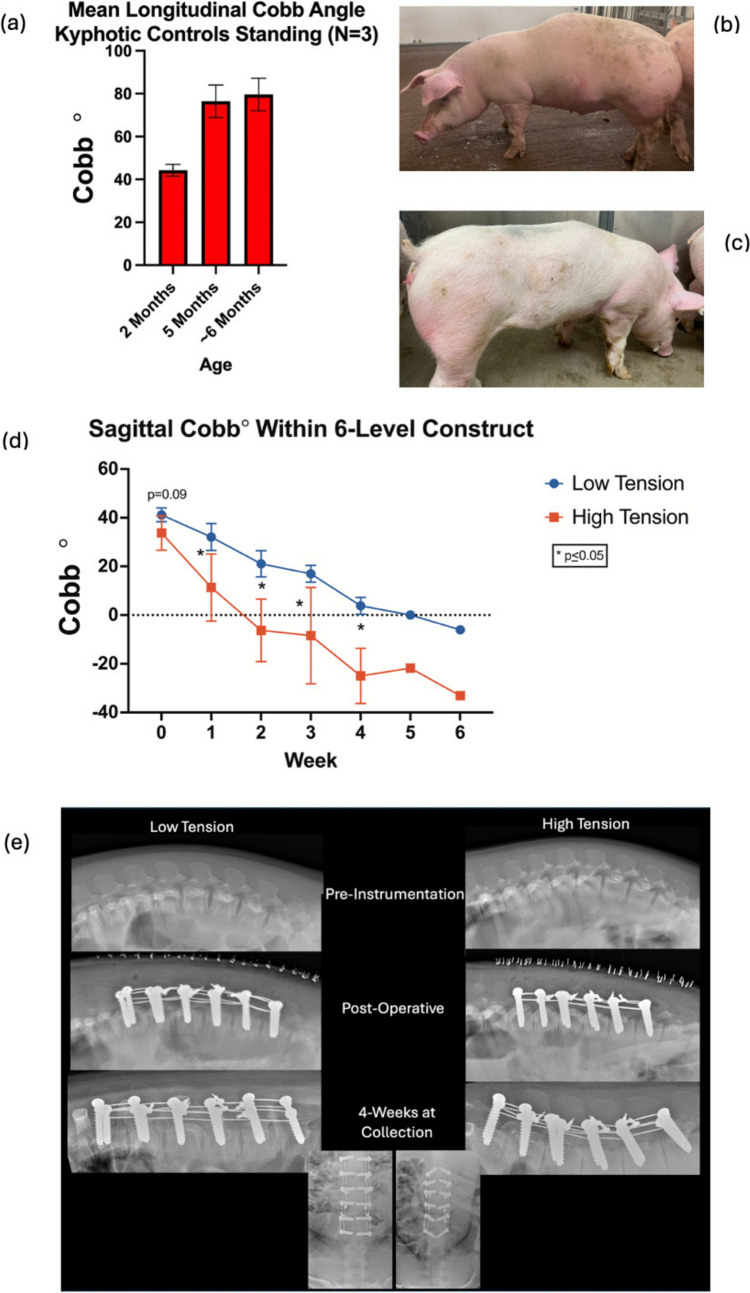
**a** Longitudinal sagittal Cobb angles of *N* = 3 untreated non-operative kyphotic swine. **b** Clinical picture of untreated kyphotic swine and **c** swine treated with multi-tether construct. **d** Cobb Angles within the constructs corrected quicker during the first 2 weeks in the high-tension group, however, the rate of correction thereafter was similar between cohorts. **e** Serial Radiographs of Low- and High-Tension Spines

The mean age of tethered animals used in growth rate measurements was slightly older than their non-operative controls (13.1 ± 0.1 vs 12.5 ± 0.3 weeks, *p* = 0.002). While significant differences in anterior and posterior growth rates between tethered and non-operative controls were found 2–4-weeks post-operative in the multi-level cohort, apical growth modulation was normalized (*p* = 0.6) between the low (14 ± 11%) and high (10 ± 10%) tensioned tethers (Fig. 4). Serial lateral Cobb measurements paralleled these growth rate findings in the multi-tether cohorts. A significantly lower instrumented Cobb angle was found in the high-tension cohort (11.3° ± 14° vs 32° ± 6° *p* = 0.04) at week one. After 2 weeks, the mean slope (degree kyphosis/week) normalized, becoming very similar between cohorts (Fig. 5). As the instrumentation did not extend the entire length of the kyphotic deformity, junctional kyphosis was observed in every animal following posterior tethering and the deformity was over-corrected into lordosis.

### Finite element analysis and growth modulation predictions

**Fig. 6:**
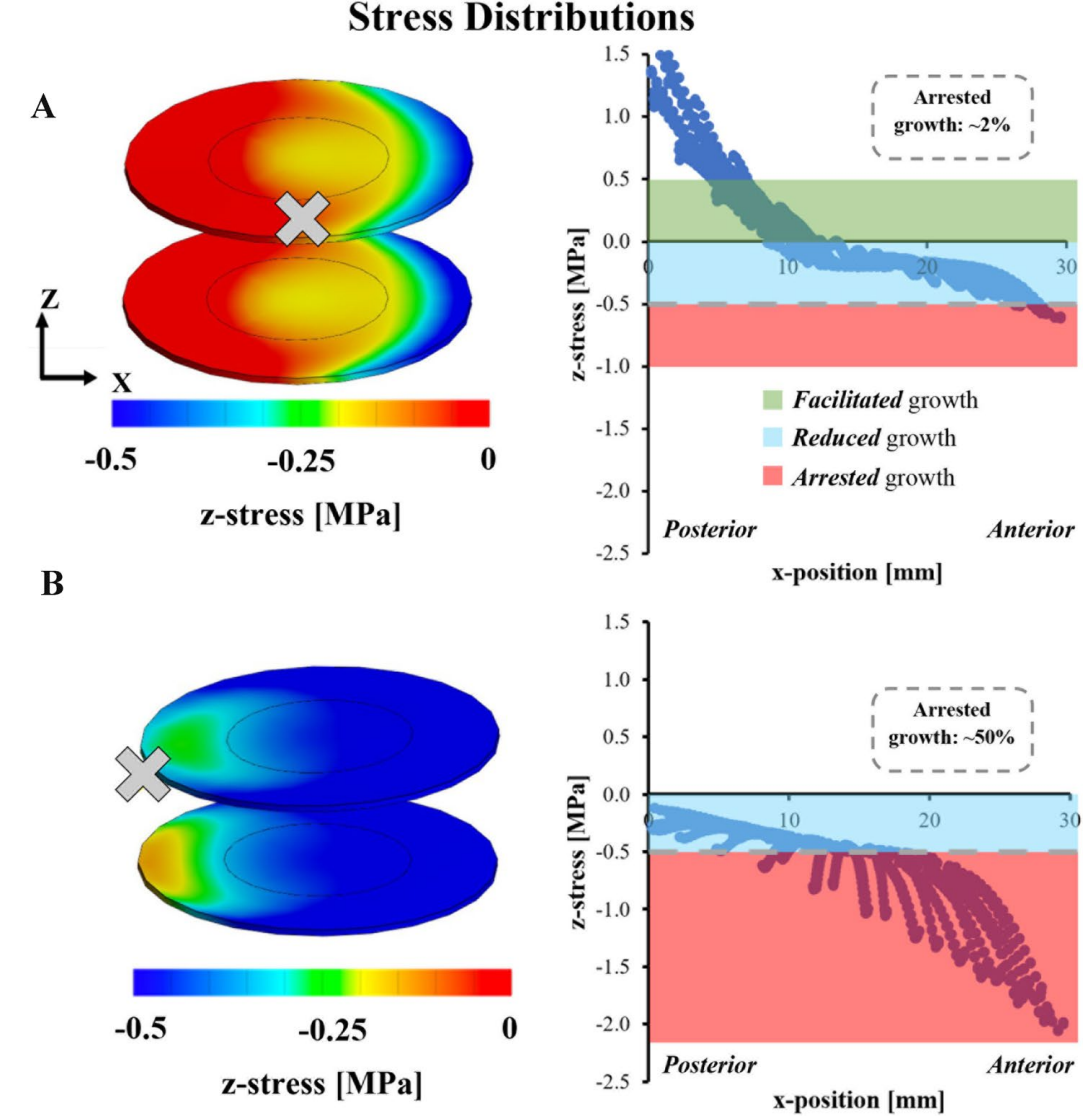
Representative growth plate mid-frontal stress distribution following a 10-degree correction through the disc space: (top row) without loss of disc height (mimicking in-vivo data in young flexible spine) or (bottom row) without gain of anterior disc height (modeling a “stiff” disc space). In both rows, left-hand side is anterior vertebra position and right, posterior (in mm of position from anterior to posterior). Note the similarity in the predicted growth modulation in the “flexible spine” with the high-tension growth rate data in Fig. 4. Conversely, note the degree of growth arrest predicted with loss of disc height (increasing disc space stiffness). 0.5 MPa and greater compressive stress was used as the threshold for based on experimental data in the literature [44, 46]

The model-predicted intradiscal pressure of 0.15 MPa agreed well with kyphotic porcine model data (0.1294 ± 0.018 MPa), and data reported in the literature (ranging from 0.1 to 0.21 MPa) [47–50]. In our model, the motion segment was subjected to flexion (torque) about the *y*-axis, combined with displacement in the *x*-axis to realign the motion segment from an angulated position (10° kyphotic) to a neutral position. Simulations of a more flexible spine showed a decrease in intradiscal pressure with tethering (0.005 MPa), which agreed with experimental observations. However, simulations of tethering in stiffer spine segments predicted an increase in intradiscal pressure (0.297 MPa). Model predictions showed that tethering procedures that maintained the mid-sagittal disc height, i.e., were flexible enough to distract anteriorly, generated a differential stress gradient (red = tensile and blue = compressive) across the vertebral growth plates (Fig. 6). The resulting predicted growth modulation curve was similar to the high tension (25.6 N) in vivo observations made in our young flexible kyphotic swine where anterior disc distraction occurred (Fig. 3). The flexibility of our young animals did not allow direct in vivo testing of conditions where mid-sagittal disc height is lost, i.e., the anterior disc cannot be distracted. Modeling under these conditions predicted significant growth arrest across the vertebral growth plate (Fig. 6).

## Discussion

The data from this study confirm that (1) the diet-induced kyphotic deformities in this model are progressive, (2) pVBT can effectively modulate vertebral growth to correct sagittal deformities, changing the natural history of the deformity, and (3) in the flexible spine, increased tether tension leads to a transitory increase in growth modulation without increasing disc pressure, but the beneficial effect is only transitory and may actually be detrimental in cases where it causes a loss of disc height (from our computational data). Classically, Stokes et al. evaluated the effects of placing varying loads across vertebral segments and measured the resultant physeal growth rates [46]. This classic study quantified the Hueter-Volkman principle, demonstrating a linear relationship between increased load and decreased physeal growth rate. However, in apparent contradiction to those findings, Newton et al. demonstrated that tether tension (i.e., load across the vertebral physis) had little effect on the subsequent rate of deformity production (beyond the initial deformation from implantation) [15]. Our study may help to shed light on this apparent contradiction. Stokes et al. evaluated the growth effects (using fluorochrome labels) of load over a brief (~ 1 week) period, whereas Newton et al. measured the radiographic changes in Cobb angle over months in response to load. Our single-level tether fluorochrome growth rate data demonstrate that in the *early post-operative period* (0–2 weeks), apical growth modulation was *load dependent*, supporting the results of Stokes et al., however, our *later* (2–4 weeks) multi-level tether data demonstrate that growth rates and radiographic changes in Cobb angle were *load independent*. Thus, taken together, these studies suggest that growth modulation may proceed through an early load-dependent phase, followed by a later load-independent phase.

Our initial hypothesis for this project was that increased tether load would cause increased disc pressures, leading to decreased overall vertebral growth and diminished growth modulation, assuming correction of the kyphotic deformity would occur through posterior compression of the disc alone, rather than a combination of posterior compression and anterior distraction. However, we found that reversal of apical disc Cobb angle from kyphotic to lordotic led to a drop in apical disc pressure with increasing posterior tether load, demonstrating the flexibility of the spines in these young animals. Clinically, the terms “flexibility” and “stiffness” of a spinal deformity often refer only to the changes in *angular vertebral alignment (Cobb angle) for a given load* or corrective measure applied to the spine. Examples include the observed change curve magnitude with upright, supine, or bending, bolster, and stretch films, or the maximal angular correction obtained in a brace or spinal implants. While perhaps subtle, in this manuscript, flexibility (of the vertebral unit) *not only refers to angular change between the vertebra, but also the effects of the load on the disc height* and therefore compression. This subtle distinction maybe fundamentally important in spinal growth modulation as FEA predicts very different vertebral growth modulating effects for the same vertebral angular correction depending on disc height. To better understand how increasing curve stiffness would have affected our growth rate findings, we adapted a validated FEM of the vertebral disc motion segment [38, 40, 42] to evaluate parameters not possible within the scope of the current in vivo work (i.e. moving the center of rotation anterior to mimic loss of disc height during correction). Using parameters of a deformed yet flexible disc, we found that the computational model predicted a stress distribution and growth modulation that mirrored the measured animal growth modulation. The FEM also supported our initial hypothesis that compressive angular correction resulting in loss of height is predicted to shut down growth, substantially inhibiting growth modulation. Future work will aim to confirm these findings in vivo.

This study used the hyperkyphotic porcine model to more accurately mimic the clinical scenario of deformity correction, rather than deformity production as previously reported [2, 13, 16]. However, this study does have some limitations. The mixed-breed swine employed here grow much faster than that reported for mini-pigs in previous VBT studies [15]. This allowed the use of pulsed-fluorochrome labels to directly measure regional vertebral growth, but limited the post-operative follow-up period to weeks, as opposed to the months or years clinically encountered. While we believe the overall response of slower-growing tethered physes would be similar, the absolute duration of each phase may differ. Similarly, the maturity of the swine used in this study would be “younger” than typical human adolescent patients. From years of swine breeding and research [28, 51–53], swine do not experience an equivalent to the human adolescent growth spurt (as their growth is more linear). As managed in our herd, pigs reach puberty between 20 and 24 weeks of age at a body weight of approximately 130 kg, a weight greater than twice that of an average adolescent human [54]. Despite the differences in growth pattern and size, if puberty is used to align our pig model with human age, the pigs in this study reflect pre-adolescent ages of 4- to 8-year-old humans based on a comprehensive review of physiological developmental similarities for various organs of swine and humans [55]. Thus, the age of the animals reported may be considered “less mature” than current clinical pVBT applications, however, the ages and anatomical features are consistent with selection swine as a translational model for orthopedic research [56]. Similarly, the range of loads applied to the tethers was limited by the screw plow in the young animals; however, a five-fold difference in load on the vertebrae was achieved and was sufficient to demonstrate a differential growth response. In following the 3R principles of animal research, we chose to compare the growth modulation of a single tethering at one post-operative period (0–2 weeks) with the apical growth modulation of a multi-tethered spine at a later period (2–4 weeks). This was a development that occurred as we became more experienced with the approach, instrumentation, and perioperative animal care after this novel model and procedure, we felt expanding our findings from the single level to a multilevel construct would be more clinically applicable. While repeating either the single-level experiments for a longer duration (2–4 weeks) or the multilevel experiments for a shorter period of time (0–2 weeks) may have minimized potential confounding factors, the authors felt the additional animal lives and costs outweighed the information gained. It is possible that the blunted effect of tether load at the later period was due to the position of the apical vertebra within the tethering construct. However, this is unlikely, as we have analyzed the growth modulating effects of vertebral position within the tethering construct and present this data in a separate accompanying manuscript. Additionally, when measuring the change in Cobb angle in the multi-tether cohorts, junctional kyphosis led to skeletal deformities within the last instrumented vertebra, and as a result the Cobb angles were measured within the construct only, as opposed to the outside of the last instrumented vertebra. Furthermore, any attempts to arbitrarily define “regions” along the vertebral endplate are artificial and prone to criticism. As the tether load is transmitted through the disc space to the physes, the regional stress is predicted to be different based on the mechanical properties of the intervening disc structure [35–41] Our 1/4, 1/2, 1/4 breakdown was anatomically based on the areas of the vertebral physis lying under the NP (~ central 1/2) versus those lying under the anterior and posterior annulus. Given variations in the annulus and NP position in individual samples and at different time points, our regional delineation may not be perfect, however, the authors felt this standardized measurement would minimize the “mixing” of central growth rates (from regions under the NP) with those at the anterior and posterior endplate and maximize our ability to detect regional differences. This decision was later supported by our post-hoc FEA modeling, which demonstrated a non-linear stress distribution across the vertebral endplate with the greatest stress gradients occurring at the vertebral edges rather than across the broad central region. We have included a separate analysis (Supplemental Materials) to demonstrate the effect regional definitions have on the ability to detect growth modulation using fluorochrome labeling. Thus, the in-vivo and FEA data suggest that vertebral growth modulation occurs primarily at the vertebral periphery and very little stress differential and, therefore, growth modulation occurring centrally. Finally, given the scope and variables tested in this work, sample size for each variable tested was limited.

In conclusion, this study demonstrates the feasibility of correcting sagittal plane deformities using posterior vertebral tethering. The data presented demonstrate that in the flexible spinal deformity, growth modulation of tethered vertebra proceeds through an early, load-dependent response that later becomes load-independent. Future studies will focus on the effects of tether tension on stiffer spines with less remaining growth, as our computational analysis predicts the results to differ from those in the flexible spine. As junctional kyphosis was observed in these animals, further investigation is warranted prior to clinical translation.

## Supplementary Information

Below is the link to the electronic supplementary material.

Supplementary file1 **Supplemental Materials. (a)** Representative fluorochrome histologic image (4X) demonstrating the relationship of annulus, NP, and physis with sagittal divisions**.** Graphical analyses were performed to compare the effects of alternative regional divisions of the vertebral physis using our single-level samples divided: **(b)** anterior and posterior halves, **(c)** thirds, **(d)** fourths (using our previous anterior and posterior ¼ measurements and dividing our previous central ½ measurement into two separate ¼ measurements). These are each plotted against our reported (¼, ½, ¼) measurements of the control (black dashed), low tension (blue dotted), and high tension (red dotted) presented in the manuscript. These graphical representations demonstrate the effect of including the central region of the vertebral physis in either the anterior or posterior regions, as this “unmodulated zone” normalizes the peripheral differences and limits the ability to detect differential growth. While our generalized FEA model does not exactly mimic each surgical sample tested (Fig. 6) it graphically demonstrates that the greatest stress gradients are found localized anteriorly and posteriorly, with very little differential stress found centrally mirroring that of our presented in vivo data or the division of the vertebra by 1/4 s. Despite being variable in size from sample to sample, the additional measurement of the “anterior unossified region” included in (Figs. 3, 4) further supports these findings as this region was found at the anterior-most border of the vertebrae and shows to have even greater modulation than that of the entire anterior ¼ vertebra. While not the primary goal of this work, these data provide novel insight as to *where* on the vertebrae growth is being modulated by a unilateral tether. In future work, better regional resolution of growth modulation could be achieved by measuring growth pixel by pixel moving from anterior to posterior and plotting it against % anterior/posterior location on the vertebral body. (TIF 16641 KB)

## Data Availability

Data is available upon reasonable request.
