# Supplementary figures and images for: The growth modulating effects of tether tension on vertebral growth are biphasic: a study of posterior vertebral body tethering (pVBT) in a novel kyphotic porcine model
Source: Spine Deform. 2025 Aug 20;14(1):19–30. doi: 10.1007/s43390-025-01168-y (PMC12815995; doi:10.1007/s43390-025-01168-y)

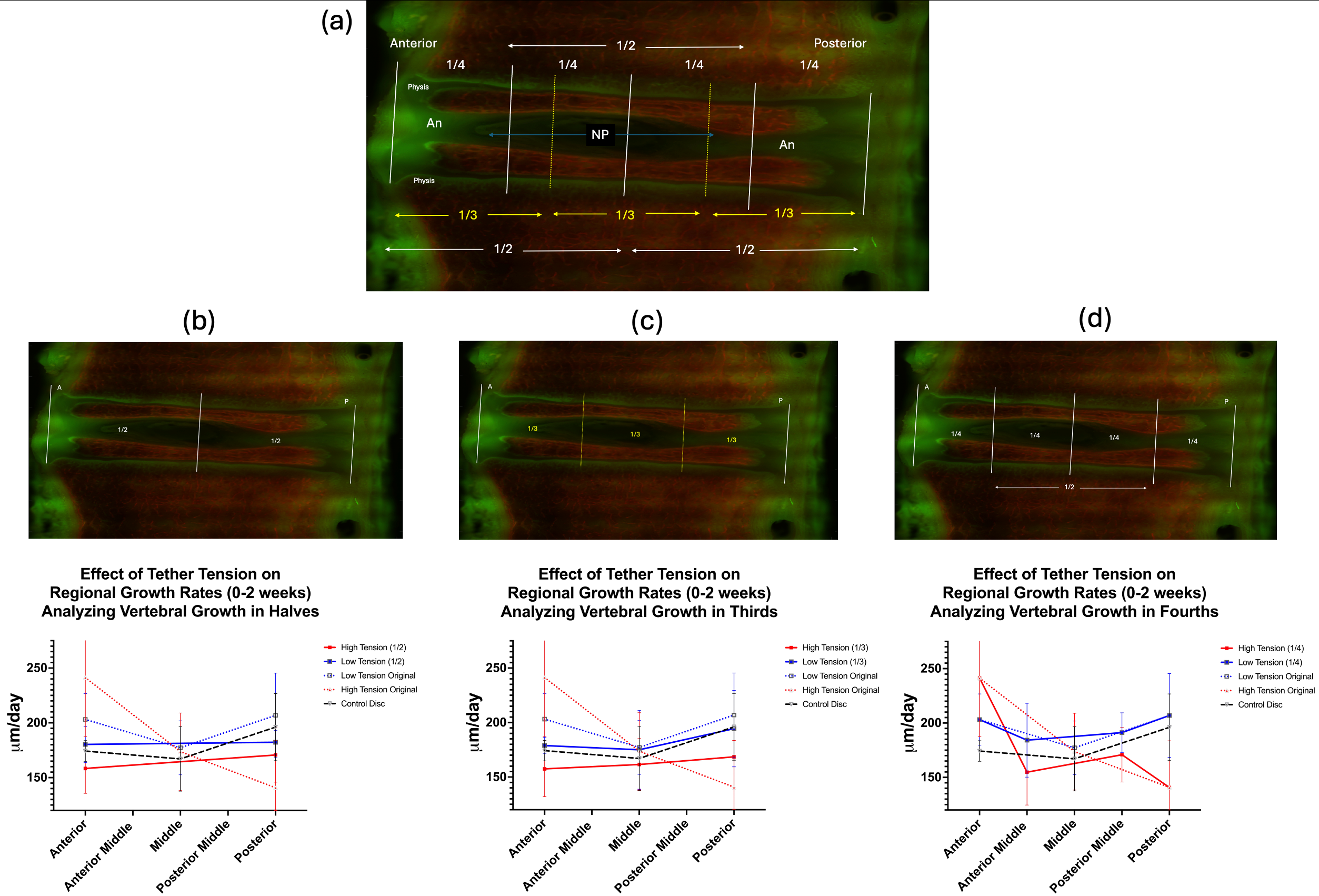

Supplement: Supplementary file 1 — Supplementary file1 Supplemental Materials. (a) Representative fluorochrome histologic image (4X) demonstrating the relationship of annulus, NP, and physis with sagittal divisions. Graphical analyses were performed to compare the effects of alternative regional divisions of the vertebral physis using our single-level samples divided: (b) anterior and posterior halves, (c) thirds, (d) fourths (using our previous anterior and posterior ¼ measurements and dividing our previous central ½ measurement into two separate ¼ measurements). These are each plotted against our reported (¼, ½, ¼) measurements of the control (black dashed), low tension (blue dotted), and high tension (red dotted) presented in the manuscript. These graphical representations demonstrate the effect of including the central region of the vertebral physis in either the anterior or posterior regions, as this “unmodulated zone” normalizes the peripheral differences and limits the ability to detect differential growth. While our generalized FEA model does not exactly mimic each surgical sample tested (Fig. 6) it graphically demonstrates that the greatest stress gradients are found localized anteriorly and posteriorly, with very little differential stress found centrally mirroring that of our presented in vivo data or the division of the vertebra by 1/4 s. Despite being variable in size from sample to sample, the additional measurement of the “anterior unossified region” included in (Figs. 3, 4) further supports these findings as this region was found at the anterior-most border of the vertebrae and shows to have even greater modulation than that of the entire anterior ¼ vertebra. While not the primary goal of this work, these data provide novel insight as to where on the vertebrae growth is being modulated by a unilateral tether. In future work, better regional resolution of growth modulation could be achieved by measuring growth pixel by pixel moving from anterior to posterior and plotting it against % a [file 43390_2025_1168_MOESM1_ESM.tif]
